# Assessment of Adult's Abdominopelvic Computed Tomography Radiation Doses in Amhara Region, Ethiopia

**DOI:** 10.4314/ejhs.v32i6.6

**Published:** 2022-11

**Authors:** Seife Teferi Dellie, Ambaye Fisehaw Tesfaw, Tesfaye Kebede Legesse

**Affiliations:** 1 Department of Radiology, College of Health Sciences, Addis Ababa University; 2 Department of Physics, College of Natural and Computational Sciences, Woldia University

**Keywords:** Computed Tomography, Local diagnostic reference levels, Computed Tomography dose index volume, dose length product, patient doses

## Abstract

**Background:**

Computed Tomography plays a priceless role for diagnostic and therapeutic purpose; however, applying an optimized Computed Tomography Technique to produce qualified image while delivering minimum radiation dose to patients is the common challenge. The main objective of this study was to establish local diagnostic reference levels for adult patients who visited abdominopelvic Computed Tomography examination.

**Methods:**

A total of 158 patients who had taken abdominopelvic Computed Tomography examination from three selectedAmhara region hospitals were investigated. Both prospective and retrospective techniques of data collection were used while collecting the data in the entire sample. Two GE - Optima Computed Tomography 540 (16 slices) and one Phillips — Brilliance (64slices), were employed during data collections. Data for patient demographics scan protocols, Computed Tomography dose descriptors and machine specifications were collected and analyzed by using SPSS software version 26.

**Results:**

The third quartile estimated computed tomography dose index volume and dose length product, which is the local Diagnostic Reference Levels, were 12 mGy and 1904 cm.mGy respectively. The investigated local Diagnostic Reference Levels of Computed Tomography Dose index volume (mGy) was comparable to other international Diagnostic Reference Levels. However, the third quartile value of dose length product (cm.mGy) was higher than other reported international Diagnostic Reference Levels.

**Conclusion:**

The values of local Diagnostic Reference Levels presented in this work can be used as a baseline upon which future dose measurements can be compared in Amhara region.

## Introduction

Computed tomography (CT) plays a priceless role for diagnostic and therapeutic purposes and its utilization has increased globally since it is used to generate 3D images of the body. Applying an optimized CT technique to produce qualified images is the major and common challenge in CT practice while delivering minimum radiation dose to the patients. Because of this reason, the variation of CT doses for patients undergoing CT examination has increased alarmingly almost all over the world, particularly in Africa, Asia, and Eastern Europe (1). Evidence has shown that these challenges are derived from equipment's related factors (image reconstruction technique, detector efficiency, collimator type, scanning mode) and under-estimating personnel towards the risk of ionizing radiations (2).

Due to the need for higher attention toward ionizing radiation, the international commission on radiation protection developed diagnostic reference levels (DRLs) which have been used for dose optimization during CT imaging procedures (3). The international commission on radiation protection (ICRP) has provided guidelines on how Diagnostic Reference Levels (DRLs) can be established by the regulatory authorities. Based on these recommendations DRLs have been prepared from the 3rd quartile value of the mean distribution of CT dose metric values(4). To enhance the opportunity for management and optimization of CT radiation doses, local diagnostic reference level values (LDRLs) should be developed at the practice level from the local survey and these values are easily reviewed (5). All CTs scanners today display patient dose descriptors, like CT dose index volume (CTDIvol) or dose length product (DLP). Recording patient dose descriptors and finding the third quartile of them allows for knowing whether the doses are below or above the recommended values (6–10). In the Amhara region, there are few numbers of medical centers equipped with CT scanners and as far as we know, no one has conducted CT DRL for patients who visited CT examination.

Hence, the main objective of this study was to establish local diagnostic reference levels (LDRLs) and thereby recommend the best possible techniques for Computed Tomography dose reduction of abdominopelvic adult patients who have taken abdomen pelvis CT examinations in the Amhara region, Ethiopia.

## Materials and Methods

**Study design**: The study was conducted in the Amhara region from June-October 2020. It utilizes both prospective and retrospective methods of data collection with a descriptive cross-sectional study design. All active Computed Tomography (CT) scanners from public specialized teaching and referral hospitals in the Amhara region were identified and checked for quality assurance certification. All selected three hospitals have large population demography, well-organized radiologists, radiology technologists, and radiographers. The Hospitals thereafter represented as (FHCSTRH), (DCSTRH), and (UOGCSTRH). All patients who visited the three governmental hospitals during the study period were the source population. All adult patients who were sent to the radiology department for abdominopelvic CT scans in the Amhara region during the study period with their dose data available were the study populations.

**Sample size and sampling technique**: A cross-sectional study design was employed and the sample size was determined based on ICRP recommendations to conduct such a study. According to ICRP (135)(11), such CT patient dose surveys should include at least 30 patients. To increase precision a minimum of 45 patients were included from each hospital summing up to 158 patients

**Data collection procedures**: Initially, self-administered data collection forms for scan parameters; CT dose describers, CT machine specification, and patient demographics were prepared in English and distributed to all three hospitals. Accordingly, quantities for assessing CT dose descriptors like CTDIvol and DLP and Scan parameters like tube current (mA), tube current modulation (mAs), peak voltage (kVp), pitch, scan range (cm), gantry rotation time (sec) and slice thickness were collected together with patient demographics of age and sex. In addition, machine specifications, manufacturer, model, installation date, number of detector rows, scanning mode, projection type, scan phases, and presence or absence of automatic exposure control (AEC) were filled in the data collection forms. Finally, the collected data were checked for their completeness, clarity, consistency, and accuracy.

**Data analysis**: The collected data were analyzed statistically using commercially available SPSS software version 26 and Microsoft excels 2016. The minimum, maximum, mean, and third quartile values of scan parameters and dose descriptions were reported. Local Diagnostic Reference Levels (LDRLs) were established from the third quartile mean value of CTDIvol and DLP values presented in the scanner and displayed on the operator console. Finally, the obtained values were compared to other international diagnostic reference values (DRLs).

**Ethical consideration**: To respect the study group's bill of rights, ethical considerations were taken into account. Clear and detailed explanations were given to the study population about the objective of the study. Any piece of information was kept confidential by not recording the names of respondents. The study was conducted after having ethical clearance from the Research and Ethics Committee of the department.

## Results

In this study, a total of 158 patients who had taken abdomen pelvis CT examination from the selected hospitals were investigated constituting 45 (28.5%) from UOGCSTRH, 62 (39.3%) from FHCSTRH, and 51 (32.3%) DCSTRH. Table 1 shows the description of CT machines used in this study. As shown in this table Slice thickness of 5mm was used while providing abdomen pelvis CT examination in all three selected examination centers.

**Table 1:** Description of CT machines used in this study

Hospitals	Manufacturer	Model	No_ of slices	Year of	Detector	Slice thickness

				Installation	Configuration	
FHCSTRH	GE -health care	Optima CT 540	16	2018	16 × 0.5	5mm
DCSTRH	GE -health care	Optima CT 540	16	2019	16 × 0.5	5mm
UOGCSTRH	Philips	Brilliance	64	2017	64 × 0.625	5mm

Table 2 shows the mean (range) value of technical factors and CT dose descriptors for each examination center. As shown in Table 2, except FHCSTRH, which uses variable pitch and constant kVp the other two were using constant pitch and kVp for the entire examinations. Table 3, gives the third quartile values, in terms of the CTDIvol (mGy), the DLP (mGy cm), for abdominopelvic CT examination of adults of this study as well as the corresponding values reported in the literature for different countries.

**Table 2:** Adult Patients: Abdomen pelvis CT: Mean ± range) value of each and overall technical factors and CT dose descriptors for all hospitals

Hospitals	Technical factors					CT dose descriptors	

	kV (constant)	mA	mAs	Scan Range(cm)	Pitch	CTDIvol (mGy)	DLP (Cm.mGy)
FHCSTRH	120	169(278-50)	107(221-27)	30(304-2.60)	1.2(1.38-.93)	12(29.98-(6.20)	1231(3084-375)
DCSTRH	120	178(437-79)	89(212-35)	31.5(44.5-13)	1.38(1.38-1.38)	11(36.4-3.83)	1531(5343-359.5)
UOGCSTRH	120	209(720-21)	108(432-1)	44.1(57.2-1)	1.25(1.25-1.25)	10.2(12.9-5.28)	1936(5623.90-313.80)
Total	120	188(720-21)	123(432-1)	36 (57-1)	1.23(1.38-.93)		

**Table 3:** Adult patients: Abdomen pelvis CT: Comparison of the third quartile of CTDIvol (mGy) and DLP (Cm.mGy) in this study with international diagnostic reference level (DRLs)

CT dose descriptors	This study	Australia Lee, K.Let.al 2020 (7)	UK Shrimpton, P et al2005 (9)	Japan Matsunaga, Y.et.al2019 (8)	USA Kanal, K.M., et a2017 (6)	Korea Yoon, S.-W., J. 2018(10)
CTDIvol	12	13	15	20	15	10
DLP	1904	600	745	1000	755	472

## Discussion

This study showed that a large variation of dose length product appears between the hospitals for patients undergoing abdominopelvic CT examination in the Amhara region. The result indicates the necessity of setting local diagnostic reference levels for each examination type to save patients from unnecessary x-ray exposures while providing CT services. In this study, the participating hospitals that carry out abdominal CT are using multiple slices CT (MSCT): Two from General Electric and one from Philips (Table1). Different researchers have indicated that, a great variation in patient doses among different radiology departments is associated with equipment and operator-related factors (12–17). Although the last two examination centers used the same CT scanner, variations of mA were investigated. This shows a variation of patient dose from CT mainly came from techniques of utilizing scan protocols. The UOGCSTRH CT has contributed the highest patient dose due to the technical factor's mA and mAs and scan length. As shown in table 2 the highest (209mA), medium (178mA), and the least (169mA) were used in UOGCSTRH, DCSTRH, and FHCSTRH respectively. Again, the highest (123mAs), medium (107mAs), and the least (89mAs) were investigated in the centers UOGCSTRH, FHCSTRH, and DCSTRH respectively. Another factor affecting patients' radiation dose is pitch. In this study, the highest pitch (1.4) was used in DCSTRH hospital; but the lowest pitch 1.3 and 1.2 were observed in UOGCSTRH and FHCSTRH respectively. Although average scan lengths in DCSTRH (31.5cm) and FHCSTRH (30cm) were comparable, the scan range (cm) reported in UOGCSTRH (44.1cm) was highest enough (Table 2). Usage of large scan length like in scanner UOGCSTRH may lead to the extension of scan range (cm) to the upper and lower direction of tissues such as lung or pelvic tissue could be included in the scan during abdomen pelvis CT examination. Consequently, variation of reported CT dose indicators is expected since their value is averaged over the entire scan range (cm) (18). The mean CTDIvol (mGy) value in UOGCSTRH was lower by 8% than the mean CTDIvol (mGy) in FHCSTRH. Surprisingly, the obtained mean DLP (cm. mGy) value in the UOGCSTRH scanner was higher by 22.2% than the obtained mean DLP value in FHCSTRH. This shows that, even though the CTDIVOL is directly associated with DLP, the usage of the highest mA, mAs, and scan length of UOGCSTRH than either DCSTRHor FHCSTRH enables it to deliver the highest DLP than the two hospitals (Table 2). As practical evidences show that, tube current (measured in milli amperes) has a significant impact on radiation dose and image quality in x-ray-based examinations (6). When the size of selected organ increases while undertaking CT examination, the magnitude of observed DLP is expected to be high (7). In general, the different brands of CT and different slice capacities, as well as CT protocols used for UOGCSTRH, could have the highest third quartile mean of DLP than other two hospitals.

The study shows that the third quartile of mean CTDIvol (mGy) for abdomen pelvis CT was comparable with (7, 10) and less than the study done in UK (9), Japan (8), and Korean (10) studies (Table 3). However, the third quartile value of DLP (cm. mGy) in this study was higher than other reported international DRLs (472–1000 cm. mGy) (6–10) (Table 3). The possible explanations for having our hospitals' higher third quartile DLP could be the usage of high scan length as opposed to other studies.

Limitations: -We need to acknowledge two potential limitations of our study. First, we admit that the CTDIvol and DLP were evaluated from the whole scan phases rather than each scan phase. Hence, no comparisons have been made between single and multiphase examinations.

Finally, the author of this manuscript recommends that the values of LDRL presented in this work are suitable to be adopted for Abdominopelvic CT examinations in Amhara regions, Ethiopia. The out put of this research can be used as a baseline upon which future dose measurements can be compared. For a scanner in UOGCSTRH that exceeded the established LDRLs, because of its high mA, mAs and scan length usage, continuous assessment of patient received dose per examination is required to optimize scan parameters and reduce patient received dose. Furthermore, a similar type of large-scale survey should be undertaken to establish national DRLs in the case of Abdominopelvic CT examinations in Ethiopia with the involvement of relevant stakeholders
